# Oral health and quality of life in the municipal senior citizen´s social clubs for people over 65 of Valencia, Spain

**DOI:** 10.4317/medoral.21305

**Published:** 2016-10-01

**Authors:** Beatriz Sáez-Prado, María-Celia Haya-Fernández, María-Teresa Sanz-García

**Affiliations:** 1 Profesora Responsable de Geriatric Dentistry. Departamento de Odontología. Facultad de Ciencias de la Salud. Universidad CEU Cardenal Herrera. Alfara del Patriarca, Valencia, Spain; 2 Profesora Responsable de Medicina Bucal y Gerodontología. Departamento de Odontología. Facultad de Ciencias de la Salud. Universidad CEU Cardenal Herrera. Alfara del Patriarca, Valencia, Spain; 3 Profesora asociada de Matemáticas. Departamento de Didáctica de las Matemáticas. Universidad de Valencia. Valencia, Spain

## Abstract

**Background:**

The world population is aging considerably. The state of elderly´s dentition is poor. Many authors agree that the oral health status influence the elderly´s quality of life.The objective of our study was to analyze the relation between the oral health status and the general health status through the quality of life of elderly people aged 65 years or more in Valencia, Spain.

**Material and Methods:**

A cross-sectional oral health survey and an oral examination have been designed to study an elderly population. Subjects: 202 adults (103 men and 99 women). Age: 65 years of age and over. Setting: Randomly selected senior citizen´s social clubs. The Oral Health Impact Profile (OHIP-14) has been used to obtain the oral health survey. Moreover, the EuroQol-5d and a Visual Analogue Scale (VAS) have been the tools to obtain the general health status. Finally, sociodemographic and oral health questions have been needed.

**Results:**

Descriptive and inferential results have been done and the main results are the following, the mean additive score of the OHIP-14 was 8.88, the mean value of the EuroQol-5d was 0.58 and of the VAS, 72.90. The OHIP-14 was consistently and significantly correlated with the index EuroQol-5d and with variables such as number of teeth, missing teeth, DMFT, dental status (being or not edentulous) and occupation. The EuroQol-5d was related to dental habits, sex, income, systemic pathologies and filled teeth.

**Conclusions:**

The oral health has a high impact on quality of life. The oral health and the general health are closely related. The oral hygiene and getting toothless influence negatively on the quality of life of elderly people.

**Key words:**Elderly, geriatric dentistry, oral health, oral hygiene, quality of life.

## Introduction

For decades, a progressive aging of the population has been happening in different parts of the world. This is due both by longer life expectancy (for medical and dietary advances) and the decrease in the birth rate. The average age of the world population will increase from 26.6 in the year 2000 to 37.3 in the 2050 and later to 45.6 in the 2100 (1).

Several authors note that Spain will be the most aged country in the 2050, since the 44.1% of the Spanish population will be older than 60 years and the average age will be 55.2 (2).

According to the municipal census on 1st January 2014 there are more than 8 million people aged 65 and over in Spain, that represents a 17.91% of the total population. Between the years 1992 and 2013, the men’s life expectancy at birth has increased from 73.9 to 80 years and women from 81.2 to 85.6, according to mortality tables published by the Spanish National Statistics Institute (INE) (3).

Valencian population characteristics do not differ from that seen in Spain and other developed countries. The proportion of over-64s in Valencia in 2014 is 17.61%, a 3.2% higher than in 1994. Being the current Aging Index 107.005%, much higher than in 1954 (76.55%) (4).

As in many Western countries, elderly Spaniards on average have a very poor economic situation (5). This can lead to the Frolich’s inequality paradox that states that there are vulnerable groups in the society with greater health needs and greater social deprivation as well, but they get poorer health care. The institutions must adapt themselves to the needs of this sector of population which is growing at such a high pace (6). In Spain the oral health improvement has not gone in parallel to the increase of elderly population (7).

The state of dentition in the elderly has repercussions on the ability to carry out daily activities, with quality of life most seriously affected with regard to eating and enjoying food (7). Numerous studies have concluded that the oral health status influence the elderly´s quality of life (7-9). But, the mouth has also other important features such as speaking, smiling or kissing, all related to the social interaction of the person.

The oral health is included in the general health and both are closely related. Therefore, to maintain a good oral health can contribute to improve the general health and thus doubtlessly the quality of life (QoL).

Oral welfare indicators emerged during the 1970s to evaluate the physical, psychological and social impact of the oral problems, and supplement the information offered by the clinical indices, since these are not responsive to subjective perceptions such as the pain, the aesthetics, the function, etc (10). In addition to mortality, morbidity, and patient satisfaction, health-related quality of life (HRQOL) is an outcome of health care as well as a consequence of illness or injury. Consequently, instruments to assess HRQOL have become important outcome measures for the evaluation of health care. The last 2 decades have seen the development of hundreds of HRQOL instruments, which are increasingly being incorporated in clinical trials (11).

The current aging figures of the elderly population, together with the general and oral health problems and with the lack of quality of life of this social group, shows the need for a better understanding of the aging process, oral health and quality of life of the people over the age of 65.

Oral problems can affect the people’s general quality of life, since the stomatognathic system is involved in critical tasks such as those listed above. In addition, periodontal disease affects or worsen other pathologies such as the osteoporosis, diabetes, cardiovascular disease or lung disease (12).

A multitude of studies (7-9,13) have been performed on the oral health and quality of life in other countries, a few in Spain (14), but Valencia lacks this type of study. It would be interesting to study. The aim of this study has been to determine if there is a relationship between the oral health status and the general health status and the quality of life of the people older than 65 in Valencia city, because this aspect has not been assessed previously.

## Material and Methods

An observational, cross-sectional, descriptive and inferential study were performed. The subjects sample has been taken from the senior citizens´ social clubs of Valencia to prevent the bias of the usual patients of a dental clinic. The study was approved by the ethics committee of the Cardenal Herrera CEU University.

Participants:

The sample size was 202 to achieve a confidence interval of 95% with a margin of error of 6.88%. The formula used was MOE = 0.98 · √ (N-n) / (N · n-n)). Where N is the population size (40.070 affiliated from the senior citizens´ social clubs of Valencia) and n the sample size and MOE is the margin of error and the 0.98 is obtained by the confidence interval of 95%. The sample size is similar to previous studies (8,13). The age of participants ranged from 65 to 88, 99 were women (49%) and 103 men (51%), (see  Table 1 for further information),

**Table 1:**
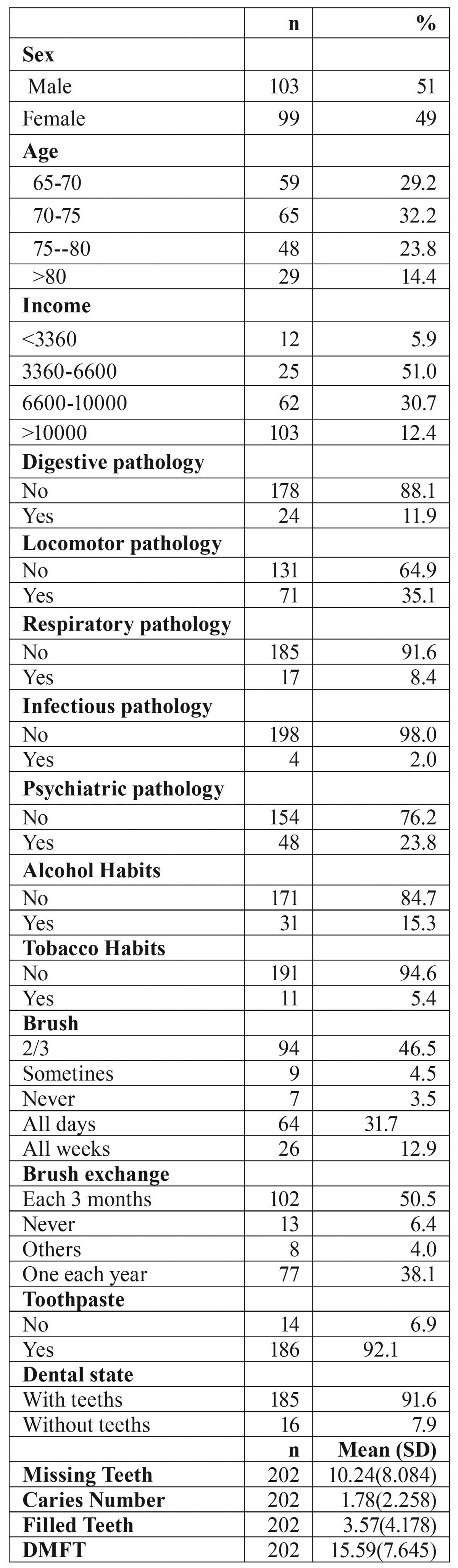
Descriptive Analysis.

The stated inclusion criteria were:

- Being older than 65 years old

- Being a member of one of the senior citizens´ social club of Valencia (Spain) selected

- Not dependent persons

- Without mental disability that prevent them from fill in the questionnaire

- That sign the informed consent 

Note that there were people who did not complete the questionnaire or the oral examination.

18 clubs from 10 districts were randomly selected, the only restriction was to have more than 700 affiliates. All the examination material was steriled in separated bags.

Data collection:

The examinations were carried out from April 2014 to June 2015. It was performed by one experienced dentist.

The questionnaires were administered during a personalized interview to ensure the proper understanding of the contents by the subjects sample. The protocol included: filiation, oral health questionnaire (OHIP-14 questionnaire (15)) and general health questionnaire (EuroQol-5d questionnaire (16)). The Oral examination included: number of teeth, DMFT, oral mucosa pathology (17), need for dental prostheses, need for dental extractions.

Statistical procedure:

The SPSS program was used to process and tabulate the information. Firstly, basic descriptive statistics were performed. Secondly, Pearson Chi-squared tests were used to obtain the posible link between qualitative variables, as in Dahl’s article (8) from Oslo University, where also was evaluated the quality of life of the elderly Norwegian. The relation between the continuous quantitative variables was analysed with the variance analysis and Spearman Rho test, as in the article mentioned above.

In all of the cases studied, *p*-value was obtained, being considered statistically significant those with value <0.05.

The intraobserver error has been 0.447214 with Dahlberg statistic, √ ( S (d-D)2/ (n-1) ) where d is the difference between the first and the second measurement, D is the average of these differences and n is the total number of individuals in the sample. 

The Kappa Cohen value was 1 to DMFT (very good concordance), 0.667 to Decayed teeth (0.6-0.8 good concordance), 1 to Missing teeth (very good concordance), 0.762 to Filled teeth (good concordance).

## Results

Results of the association between the OHIP-14, EuroQol-5d and VAS with the quantitative variables analysed:

It was analysed the possible link between the discrete quantitative variables existing in the study (caries, missing teeth, filled teeth and DMFT) and the variable OHIP-14, EuroQol-5d and VAS using Pearson’s correlation. It is observed that, Missing teeth and DMFT have been related with OHIP-14 (*p*-value-value <0.05 (level of significance)), and Filled teeth is related with EuroQol-5d ( Table 2).

**Table 2:**
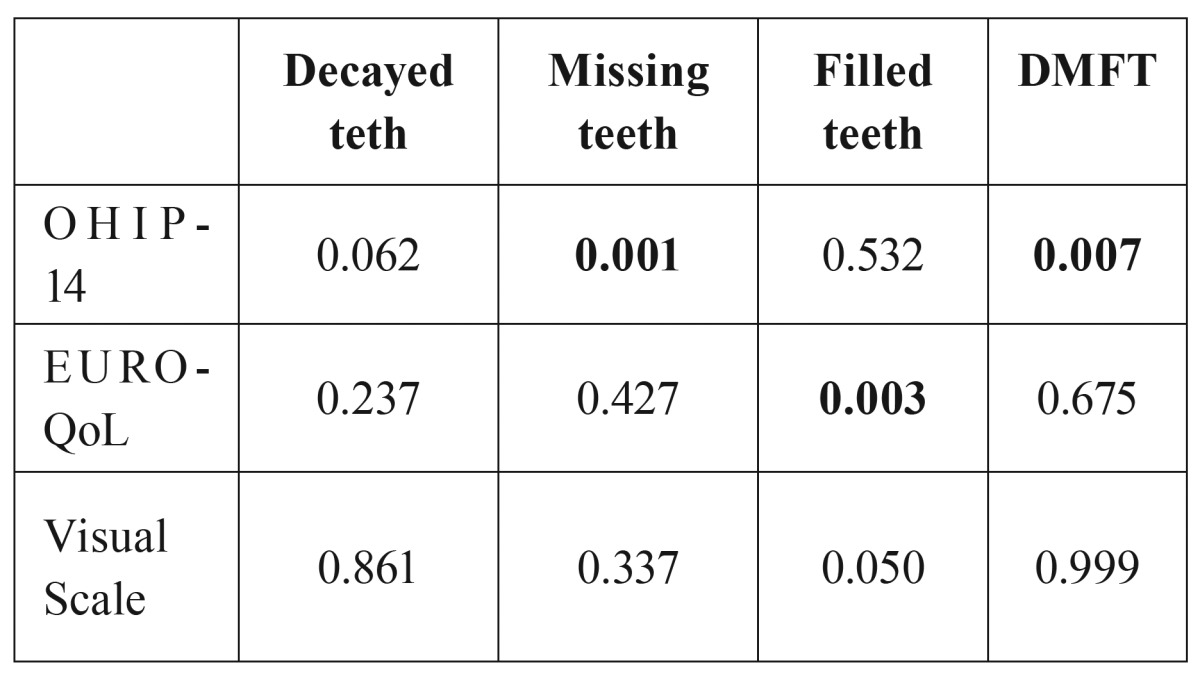
Link between OHIP-14, EURO-QoL and the visual analogue scale with decayed , missing and filled teeth and DMFT. Spearman Rho test.

The three basic quantitative variables, which are EuroQol-5d, OHIP-14 and the VAS figures, are related. Note the relation between EuroQol-5d and VAS and EuroQol-5d and OHIP-14 (*p*-value<0.05). This indicate that the respondents’ answers to both indices of general health have been consistent. Furthermore, it appears that there is also a relation between oral health and general health related quality of life HRQoL.

Results of the association between the OHIP-14, EuroQol-5d and VAS with the qualitative variables analysed:

After perform Pearson Chi-squared test, Occupation, Number of teeth and Dental State are related with OHIP-14. In the case of VAS, the variables which are related with it are Occupation and Alcohol habits. Finally, in the case of EuroQol-5d, there are more variables, for example the Sex, Income or dental habits and pathologies (detailed in  Table 3).

**Table 3:**
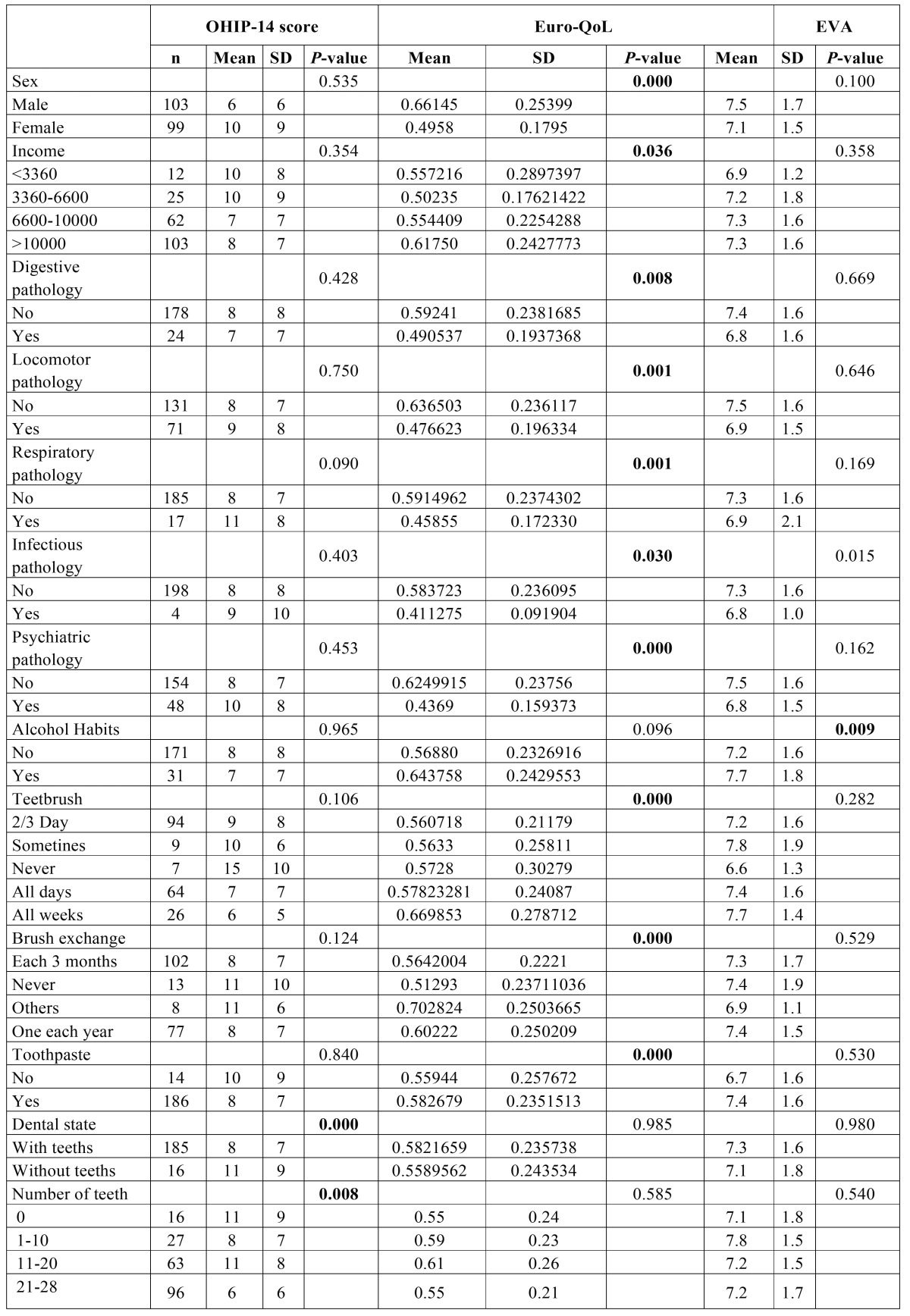
Link between the qualitative variables and the OHIP-14. EURO-QoL and the visual scale. *p*-value < 0.05. Test Pearson’s Chi-Square.

## Discussion

- OHIP-14, EuroQol-5d and VAS results and their relation 

Hebling E and Pereira AC (18) concluded that Geriatric Oral Health Assessment Index, Subjective Oral Health Status Indicators, Oral Health Impact Profile-49, Dental Impact on Daily Living, Oral Health Impact Profile-14, Oral Impact on Daily Performances and German Version of the Oral Heath Impact Profile were considered as instruments of choice to assess oral health related quality of life (OHRQoL) in the elderly. For this reason, the Oral Health Impact Profile-14 has been chosen to evaluate OHRQoL.

The average of the total count of OHIP-14 was 8.88, it is quite different from that on the previous researches, Motallebnejad *et al.* (19) 22.4, Frohlich and Potvin (6) 15 and Dahl *et al.* (8) 3.4. Nevertheless, the results here were similar to the obtained by Kotzer *et al.* (20) in Canada and Dahl *et al.* (8) in Norway. Both carried out a research on sampled elders and pointed out that the dimensions with higher impact were: psychological discomfort and physical pain, in the same way that in this study. The categories of lower impact were social inability and handicap, this aspect can be explained because people with these characterisitics can hardly attend these clubs.

The average of EuroQol-5d was 0.58 ± 0.235, slightly lower than the result obtained in the study carried out in Valladolid by Morchón-Simón *et al.* (21) that was 0.63± 0.21, despite it was performed in chronic obstructive pulmonary disease (COPD) patients.

The VAS average was 72.90±16.019. This value is similar to the value obtained in a study performed in Korea, that was 74.3 with a standard deviation of 17.8 (22).

Two important results in this study were the relation between EuroQol-5d index and the VAS (*p*-value 0.000), also there was a significant relation between OHIP-14 and EuroQol-5d index (*p*-value 0.003). Barrios *et al.* (23) find a significant association between long-term OHRQoL and HRQoL in oral and oropha-ryngeal cancer patients using 12-Item Short Form Health Survey (SF-12), the Oral Health Impact Profile (OHIP-14) and the Oral Impacts on Daily Performances (OIDP).

Oral examination variables related OHIP-14, EuroQol-5d and VAS

Statistically significant relation was found between number of teeth, missing teeth, DMFT and dental state (be or not edentulous) and the OHIP-14.

The number of teeth had a significant relation with the quality of life, also, being toothed or toothless did have relationship with the QoL (24). It seems that being totally or partially toothless severely affects the elderly people from Valencia. Another reason can be that there is a large variation between the retention of a removable denture when there is or not a residual tooth in the mouth. This can affect the chewing and as a result the quality of life. A Brazilian study published in 2012 by Rodrigues *et al.* (25) regarding the QoL and the edentulism in elders confirms this view. They used the questionnaire WHOQOL-Old and found a significant relationship between the social participation and the edentulism, supporting therefore the negative impact of being toothless in the social aspects.

In this sense, Rebelo *et al.* (26) wrote about higher income was linked to better dental status, which was linked to better OHRQoL using GOHAI questionnaire.

Whereas that, Santucci and Attard (27) found a significantly associated between oral health-related quality of life with pocket depth, decayed, missing, or filled teeth, carious teeth, number of missing teeth and types of prostheses (OHIP-14 and GOHAI, *p*<.05); and maxillary and mandibular dentures (OHIP-14 and GOHAI, *p*<.0001).

Mozafari *et al.* (28) found that ill-fit dentures, oral ulcers, pain and tooth mobility were major determinants of OHR-QoL using Oral Impact of Daily Performance.

In this study, statistically significant relation has been found between the use of teethbrush, the use of toothpaste and the brush exchange with EuroQol-5d. Kumar *et al.* (29) made a study to determine the relation of life style with dental health behavior such as tooth brushing frequency, use of extra cleansing devices and regular visits to dentist among rural residents of Udaipur district, India. Logistic regression analysis revealed that female and subjects with good current mental health status were more regular in brushing their teeth and using extra cleansing devices.

The toothbrush and the toothpaste were related to the QoL at an oral level. It seems that the hygiene habits remain the prevention bedrock of the oral pathologies, and using the brush and the toothpaste can improve our perception of the oral quality of life.

Other variables related OHIP-14, EuroQol-5d and VAS

Occupation had a statistically significant relation with OHIP-14 and VAS. Kumar *et al.* (29) found that occupation showed relation with brushing frequency, regular dentist visits and use of extra cleansing devices; housewives were more regular in brushing their teeth (OR=1.51) and using extra cleansing devices as compared to other occupation groups.

The OHIP-14 results did not have significant relationship with the level of education, this is in line with the Dahl *et al.* study (8), where they still found no relationship between the schooling and the OHIP average. The gender, income, existence of certain systemic pathologies or unhealthy habits did not obtain significant relationship with the OHIP-14, all the same the relation with EuroQol-5d was significant. The study made by Kumar *et al.* (29) subjects who suffered from systemic disease showed negative association with use of extra clean-sing devices but showed positive association with regu-lar visits to dentist.

Rebelo *et al.* (26) concluded that being older predicted lower schooling but higher income. Higher income was linked to better dental status, which was linked to better OHRQoL. Income was associated with dental clinical status via education, and income predicted OHRQoL via education and clinical measures.

Finally, alcohol habit and VAS was linked. According to Kumar *et al.* (29) subjects who consumed alcohol were less habitual for regular visits to dentist and less used to all the oral hygiene habits.

## Conclusions

The oral health has a high impact on quality of life. The oral health and the general health are closely related. The oral hygiene, missing teeth and getting toothless influence negatively on the quality of life of elderly people.
